# Tissue plasminogen activator disrupts the blood-brain barrier through increasing the inflammatory response mediated by pericytes after cerebral ischemia

**DOI:** 10.18632/aging.102431

**Published:** 2019-11-18

**Authors:** Eryan Yang, Ying Cai, Xiuhua Yao, Ji Liu, Qixue Wang, Weili Jin, Qiaoli Wu, Weijia Fan, Lina Qiu, Chunsheng Kang, Jialing Wu

**Affiliations:** 1 Graduate School of Tianjin Medical University, Tianjin 300070, China; 2 Tianjin Key Laboratory of Cerebral Vascular and Neurodegenerative Diseases, Tianjin Neurosurgical Institute, Tianjin Huanhu Hospital, Tianjin 300350, China; 3 Department of Neurology, Tianjin Huanhu Hospital, Tianjin 300350, China; 4 Key Laboratory of Neurotrauma, Variation, and Regeneration, Ministry of Education and Tianjin Municipal Government, Tianjin Neurological Institute, Department of Neurosurgery, Tianjin Medical University General Hospital, Tianjin 300052, China

**Keywords:** blood-brain barrier, cerebrovascular disease, inflammation, pericyte, tPA

## Abstract

Pericytes, important elements of the blood-brain barrier (BBB), play critical roles in maintaining BBB integrity and modulating hemostasis, angiogenesis, inflammation and phagocytic function. We investigated whether pericytes are involved in the recombinant tissue plasminogen activator (rt-PA)-induced inflammatory response, which disrupts the BBB, and investigated the potential mechanisms. Middle cerebral artery occlusion (MCAO) and oxygen-glucose deprivation (OGD) were employed to mimic hypoxic-ischemic conditions. Rt-PA was intravenously injected into mice 1 h after 1 h MCAO, and Rt-PA was added to the culture medium after 4 h OGD. Rt-PA treatment aggravated the disruption of the BBB compared with hypoxia treatment, and etanercept (TNF-α inhibitor) combined with rt-PA alleviated the rt-PA-induced BBB disruption *in vivo* and *in vitro*. Rt-PA treatment increased the TNF-α and MCP-1 levels and decreased the TGF-β, p-Smad2/3 and PDGFR-β levels compared with hypoxia treatment *in vivo* and vitro. TGF-β combined with rt-PA decreased TNF-α and MCP-1 secretion and alleviated BBB disruption compared with rt-PA; these changes were abrogated by TPO427736 HCL (a TGF-β/p-Smad2/3 pathway inhibitor) cotreatment *in vitro*. Rt-PA did not decrease TGF-β and p-Smad2/3 expression in PDGFR-β-overexpressing pericytes after OGD. These findings identify PDGFR-β/TGF-β/p-Smad2/3 signaling in pericytes as a new therapeutic target for the treatment of rt-PA-induced BBB damage.

## INTRODUCTION

Ischemic stroke is the leading cause of disability and the second most common cause of mortality worldwide [1, 2]. Recombinant tissue plasminogen activator (rt-PA) has been approved by the Food and Drug Administration as a treatment for patients with acute ischemic stroke. However, in addition to the thrombolytic function of rt-PA, a growing body of evidence indicates that rt-PA treatment increases the permeability of the blood-brain barrier (BBB) [3, 4]. Ischemic stroke increases the permeability of the BBB, resulting in the development of cerebral edema. Moreover, the increased incidence of intracranial hemorrhage in acute ischemic stroke patients who received rt-PA treatment was closely associated with an increase in BBB permeability.

Many studies have suggested that inflammatory responses increase the disruption of the BBB [5, 6]. Previous studies have shown that rt-PA treatment could induce inflammatory responses after ischemic stroke [7, 8]. Pericytes, an important component of the neurovascular unit (NVU), maintain the integrity of the BBB. Pericytes are multifunctional cells that surround endothelial cells and contact many parenchymal brain cells. Studies have shown that pericytes contribute to the brain inflammatory response by generating cytokines, chemokines and adhesion molecules [9–11]. rt-PA was shown to be toxic to pericytes [12] and can affect the function of pericytes through several mechanisms, such as the detachment of pericytes from astrocytes, a reduction in pericyte coverage of endothelial cells and a reduction in neurotrophic factor secretion [13]. However, few studies have examined the effects of the rt-PA-induced inflammatory response on pericytes. A previous study showed that pericytes can increase TNF-α and MCP-1 expression after monomeric α-synuclein treatment [14]. Here, we studied the effects of rt-PA on BBB integrity and investigated the underlying mechanism of inflammation in brain pericytes.

Platelet-derived growth factor receptor-β (PDGFR-β) is a homodimeric or heterodimeric cell surface tyrosine-kinase receptor [15]. PDGFR-β and its ligands, PDGF-B and PDGF-D, play critical roles in the migration and proliferation of mesenchymal cells [16]. PDGFR-β was shown to be highly and specifically expressed in brain pericytes but not in brain microvascular endothelial cells [17]. Oxygen-glucose deprivation (OGD) and cerebral ischemia upregulate PDGFR-β expression [18]. A recent study indicated that PDGFR-β signaling plays an protective role in the recovery of BBB function and integrity after cerebral ischemia [17, 18]. PDGFR-β deletion decreased transforming growth factor-β1 (TGF-β) expression after ischemic stroke [19]. TGF-β production in brain pericytes could protect the BBB [20].

The TGF-β signaling pathways trigger many cellular processes. TGF-β is a pleiotropic factor that plays a critical role in cell inflammation, differentiation, proliferation and survival and scar formation [9, 21–23]. TGF-β ligand-binding to TGF-β receptors (TGF-βRs) initiates signal transduction pathways mainly through Smad [24]. A previous study reported that TGF-β production in brain pericytes could protect the BBB [20, 25]. In addition, the TGF-β pathway can suppress the secretion of many inflammatory factors from pericytes [9].

Based on the results of our study, we propose that rt-PA disrupts the BBB by increasing the generation of inflammatory factors in pericytes via PDGFR-β/TGF-β/p-Smad2/3 signaling after ischemia/reperfusion (I/R). The mechanism underlying this effect suggests a novel and potential therapeutic target.

## RESULTS

### Rt-PA treatment disrupts the BBB *in vivo* after I/R

**Figure 1:**
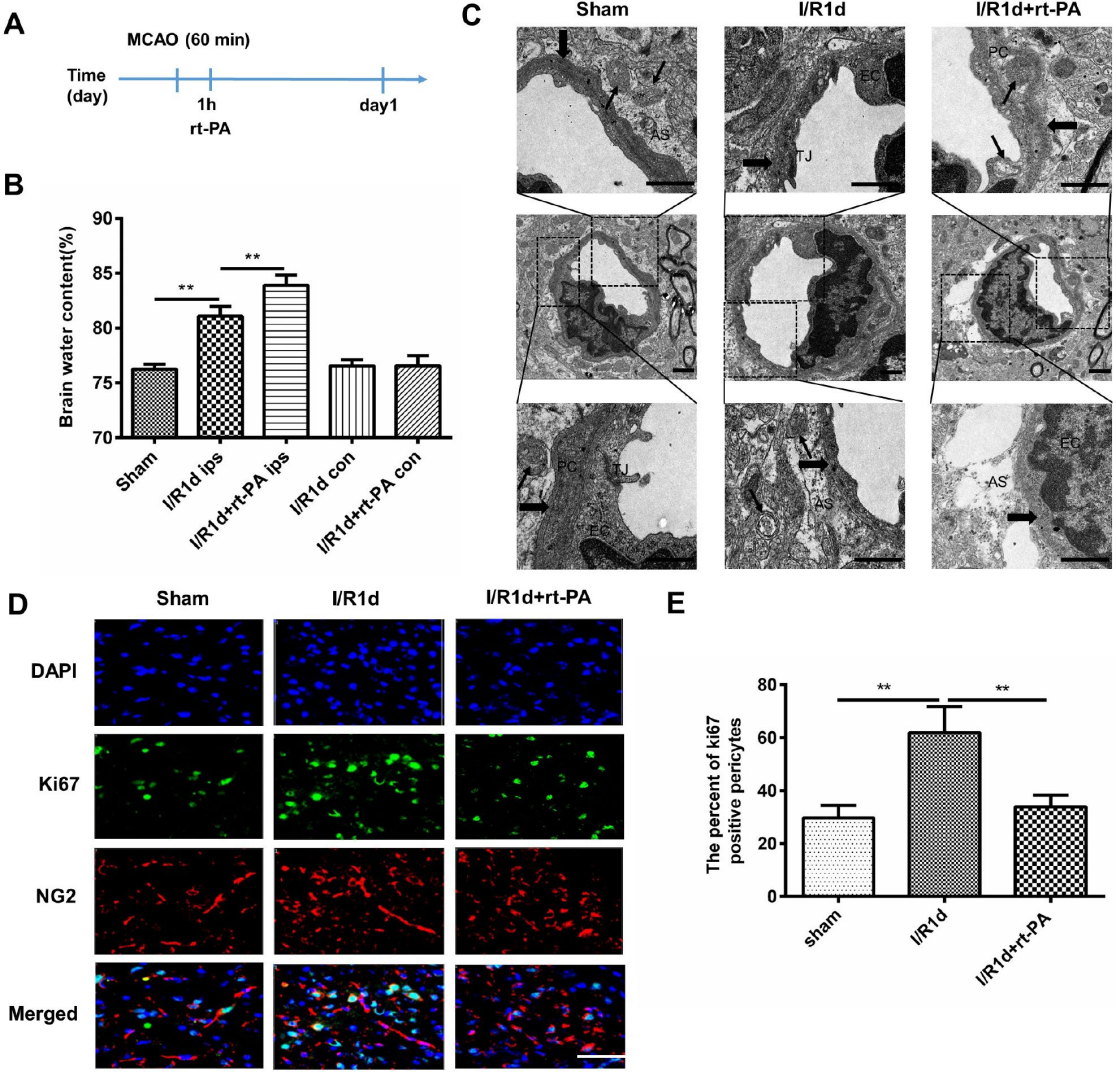
**Rt-PA treatment disrupted the BBB *in vivo* after I/R.** (**A**) Schematic of the animal study design. (**B**) Water content of the ipsilateral hemispheres and contralateral hemispheres of the brains of mice treated with or without 9 mg/kg rt-PA 1 d after I/R; ips: ipsilateral hemisphere; con: contralateral hemisphere; n = 6 for each group. Data represent the mean ± sd; *p < 0.05, **p < 0.01. (**C**) Electron microscopy was used to study the BBB ultrastructure of the sham-treated mice and mice 1 d after I/R treatment with or without 9 mg/kg rt-PA; scale bar: 4 μm. PC: pericyte, EC: endothelial cell; As: astrocyte; TJ: tight junction. The thin arrow indicates mitochondria; the thick arrow indicates the basement membrane. (**D**, **E**) Representative immunofluorescence images of Ki-67 protein expression in the pericytes of the sham-treated mice and mice 1 d after I/R treatment with or without 9 mg/kg rt-PA; scale bar: 50 μm; n = 3 for each group. Data represent the mean ± sd *p < 0.05, **p < 0.01.

Rt-PA is an important medical treatment for ischemic stroke. To study the effect of rt-PA on the BBB after I/R, we intravenously injected rt-PA into C57 mice 1 h after 1 h of middle cerebral artery occlusion (MCAO). A schematic of the animal study protocol is shown in Figure 1A. To determine the effect of rt-PA on the BBB after I/R, we assessed BBB disruption by measuring the water content of the ipsilateral hemisphere and contralateral hemisphere of the mice at 1 d after the sham, I/R, and I/R with 9 mg/kg rt-PA treatments. The administration of rt-PA increased the water content of the ipsilateral hemisphere of the brain compared with that in the I/R group, but there was no significant increase in the water content of the contralateral hemisphere (Figure 1B). In addition, we observed the BBB ultrastructure with an electron microscope after 1 d of treatment with or without 9 mg/kg rt-PA after I/R. Compared with those in the sham group, the tight junctions (TJs) tended to be looser, the astrocytes were swollen and the basement membrane was discontinuous after I/R. Rt-PA treatment following I/R induced endothelial mitochondrial edema, the disappearance of the mitochondrial cristae and aggravated astrocyte swelling compared with I/R treatment alone (Figure 1C). As pericytes are critical element of the BBB, we wanted to determine the effect of rt-PA on pericytes. According to immunofluorescence analysis, Ki-67 (green) staining in NG2 (red)-positive pericytes was decreased in the 9 mg/kg rt-PA-treated mice compared with that in the mice treated with I/R alone (Figure 1D, 1E). These results indicated that rt-PA decreases pericyte proliferation.

### Rt-PA disrupts the BBB *in vitro* after OGD/R

**Figure 2:**
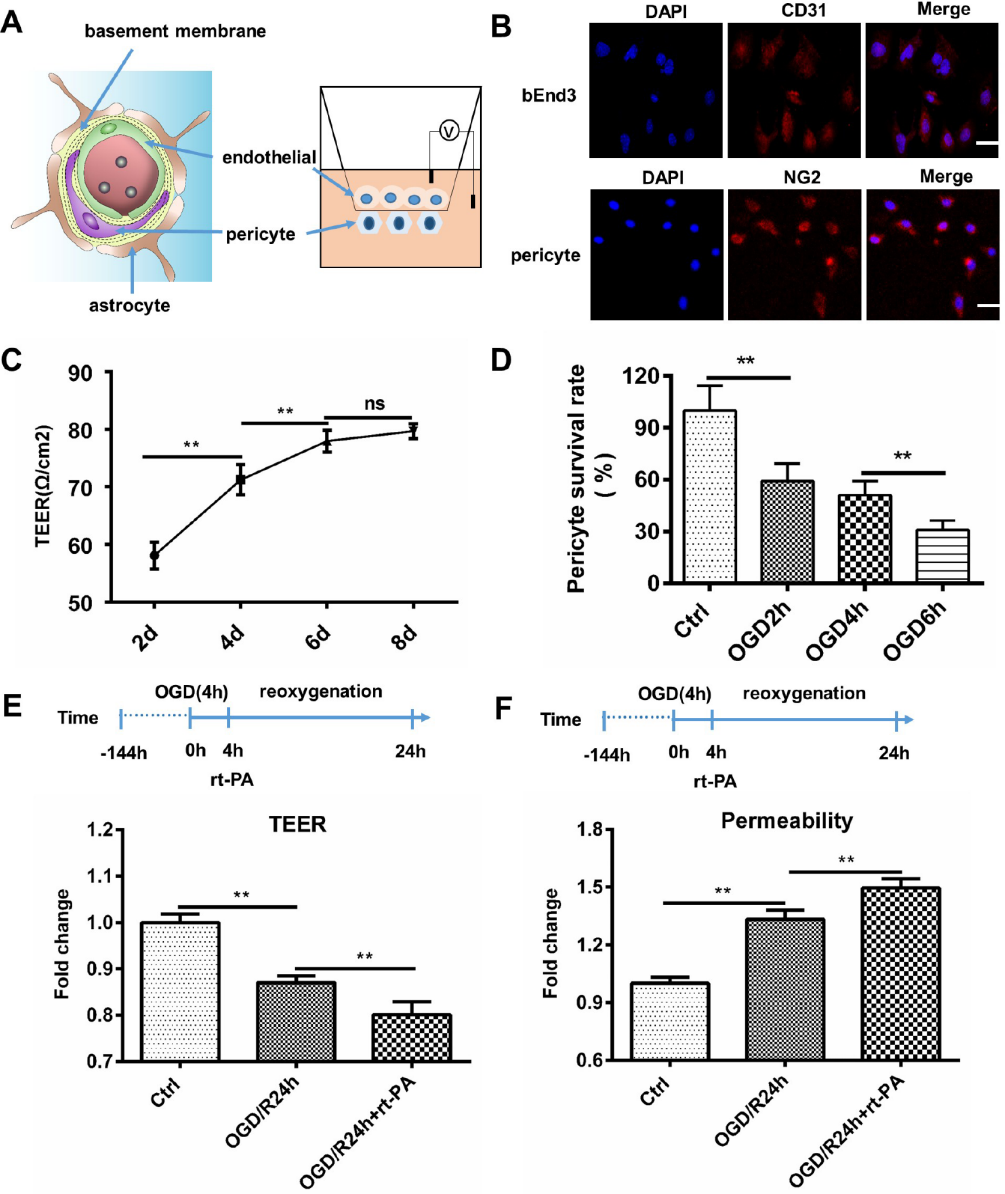
**Rt-PA disrupted the BBB *in vitro*.** (**A**) Schematic of the *in vitro* BBB model. (**B**) Endothelial cells were stained with CD31, and pericytes were stained with NG2; scale bar: 50 μm. (**C**) We determined the TEER at 2 d, 4 d, 6 d and 8 d after construction of the *in vitro* BBB model; n = 5 for each group. Data represent the mean ± sd *p < 0.05, **p < 0.01. (**D**) Mean pericyte survival was measured after OGD for 2 h, 4 h, or 6 h; n = 6 for each group. Data represent the mean ± sd *p < 0.05, **p < 0.01. (**E**, **F**) We measured the TEER and permeability of the sham-treated mice and mice 1 d after OGD/R with or without 50 μg/ml rt-PA treatment in the *in vitro* BBB model; n = 3 for each group. Data represent the mean ± sd, *p < 0.05, **p < 0.01.

To study the effect of rt-PA on the BBB after hypoxia *in vitro*, we cocultured endothelial cells and pericytes to construct an *in vitro* BBB model. We further validated the disruption of the BBB following rt-PA treatment under hypoxic conditions. A schematic of the *in vitro* BBB model is shown in Figure 2A. Endothelial cells and pericytes were used to construct an *in vitro* BBB model. Endothelial cells were stained with CD31, and pericytes were stained with NG2 (Figure 2B). The transepithelial electrical resistance (TEER) and permeability reflect the integrity of the *in vitro* BBB model. The TEER of the cocultured endothelial cell and pericyte model was increased after 2 d, 4 d, and 6 d, but the TEER after 8 d was not significantly different from that after 6 d (Figure 2C). Therefore, we chose 6 d as the time point for further analyses of the *in vitro* BBB model. To mimic *in vivo* I/R, we subjected the *in vitro* coculture model to OGD/reoxygenation (OGD/R). To determine the OGD time, we measured the pericyte survival rate after 2 h, 4 h, and 6 h of OGD. The pericyte survival rate decreased to 59.14 ± 14.39%, 50.99 ± 8.10%, and 31.05 ± 5.38%, respectively (Figure 2D). According to the survival rates, we chose to use 4 h of OGD in the subsequent experiments. To further investigate the effect of rt-PA on the BBB, we measured the TEER and permeability of the *in vitro* BBB model subjected to OGD/R for 1 d treated with or without 50 μg/ml rt-PA. The TEER decreased and the permeability to fluorescent dextran increased after OGD/R. The administration of rt-PA further decreased the TEER and increased the permeability of fluorescent dextran after OGD/R (Figure 2E, 2F). These results suggested that rt-PA disrupted the *in vitro* BBB after OGD/R.

### Rt-PA increases inflammatory factor secretion from pericytes after I/R

**Figure 3:**
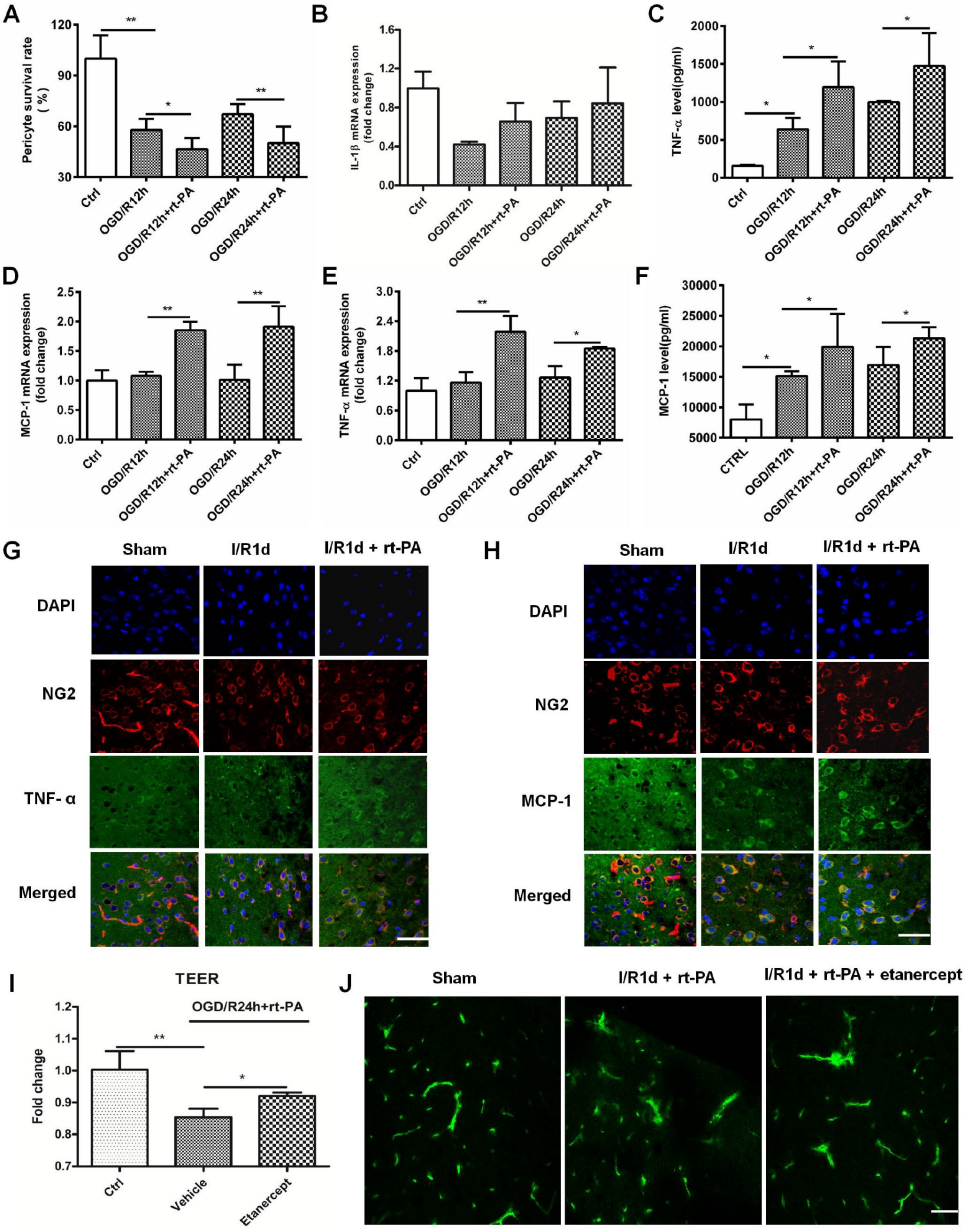
**Rt-PA increased inflammatory factor expression on pericytes after OGD/R.** (**A**) Pericyte survival was measured 12 and 24 h after reoxygenation following OGD for 4 h with or without 50 μg/ml rt-PA treatment; n = 6 for each group. Data represent the mean ± sd *p < 0.05, **p < 0.01. (**B**–**D**) The mRNA expression of IL-1β, TNF-α and MCP-1 was determined after OGD and reoxygenation for 12 h and 24 h with or without treatment with 50 μg/ml rt-PA; n = 4 for each group. Data represent the mean ± sd, *p < 0.05, **p < 0.01. (**E**, **F**) The concentrations of TNF-α and MCP-1 secreted from the pericytes after OGD and reoxygenation for 12 h and 24 h with or without 50 μg/ml rt-PA treatment; n = 3–4 for each group. Data represent the mean ± sd, *p < 0.05, **p < 0.01. (**G**, **H**) Immunofluorescence was used to detect the expression of TNF-a and MCP-1 on pericytes in the sham-treated mice and mice at 1 d after I/R treated with or without 9 mg/kg rt-PA; scale bar: 50 μm; n = 3 for each group. Data represent the mean ± sd, *p < 0.05, **p < 0.01. (**I**) The TEER were measured at 1 d after treatment with 50 μg/ml rt-PA or 40 μg/kg etanercept in combination with 50 ug/ml rt-PA after OGD/R; n = 3 for each group. Data represent the mean ± sd, *p < 0.05, **p < 0.01. (**J**) The leakage of FITC-dextran in the brains was measured at 1 d after treatment with 9 mg/kg rt-PA or 200 μg/kg etanercept in combination with 9 mg/kg rt-PA after I/R treatment; scale bar: 50 μm; n = 3 for each group.

A previous study showed that pericytes can increase TNF-α and MCP-1 expression after the administration of monomeric α-synuclein [14]. To study the inflammatory factor expression on pericytes after rt-PA administration, we first calculated the survival rate of pericytes treated with or without 50 μg/ml rt-PA at the moment after OGD for 4 h and reoxygenation for 12 h or 24 h. The pericyte survival rate decreased from 57.92 ± 6.69% and 67.27 ± 5.90% after OGD/R for 12 h and 24 h, respectively, to 46.52 ± 6.65% and 50.15 ± 9.80%, respectively, when the cells were treated with rt-PA (Figure 3A). Then, we measured the expression of IL-1β, TNF-α and MCP-1 after reoxygenation for 12 h and 24 h after OGD for 4 h. RT-QPCR was used to measure the IL-1β, MCP-1 and TNF-α mRNA levels in the pericytes after OGD and reoxygenation for 12 h and 24 h with or without 50 μg/ml rt-PA (Figure 3B–3D). The results indicated that the IL-1β mRNA expression was not significant in the pericytes after reoxygenation for 12 h and 24 h in the presence of rt-PA compared to that in the absence of rt-PA. ELISA was used to measure TNF-α and MCP-1 secretion from the pericytes after OGD followed by reoxygenation for 12 h and 24 h with or without 50 μg/ml rt-PA treatment (Figure 3E, 3F). These results indicated that the MCP-1 and TNF-α levels were increased in the pericytes after reoxygenation for 12 h and 24 h in the presence of rt- PA compared to that in the absence of rt-PA. To test whether rt-PA increased inflammatory factor expression in the mice after ischemia, we performed double-labeling immunofluorescence in the mice subjected to I/R for 1 d treated with or without 9 mg/kg rt-PA. The results of this experiment also showed that rt-PA treatment increased TNF-α and MCP-1 (green) colocalization with NG2 (red) compared with that following I/R alone for 1 d (Figure 3G, 3H). To test whether the inflammatory response participates in BBB disruption after rt-PA administration, we also measured BBB integrity *in vivo* and *in vitro* after etanercept (TNF-alpha blockers) administration. We assessed BBB disruption by measuring the TEER at 1 d after treatment with 50 μg/ml rt-PA and 40 μg/ml etanercept in combination with 50 μg/ml rt-PA after OGD/R treatment *in vitro* (Figure 3I). The results showed that etanercept in combination with rt-PA increased the TEER compared with that in the rt-PA administration alone group. We also assessed BBB disruption by measuring the leakage of FITC-dextran in the brains of mice at 1 d after treatment with 9 mg/kg rt-PA and 200 μg/kg etanercept in combination with 9 mg/kg rt-PA after I/R *in vivo* (Figure 3J). The results suggested that etanercept in combination with rt-PA decreased the leakage of FITC-dextran in the ipsilateral hemisphere of the brain compared with that in the rt-PA group.

### rt-PA-induced disruption of the BBB is mediated by PDGFR-β on the pericytes after I/R

**Figure 4:**
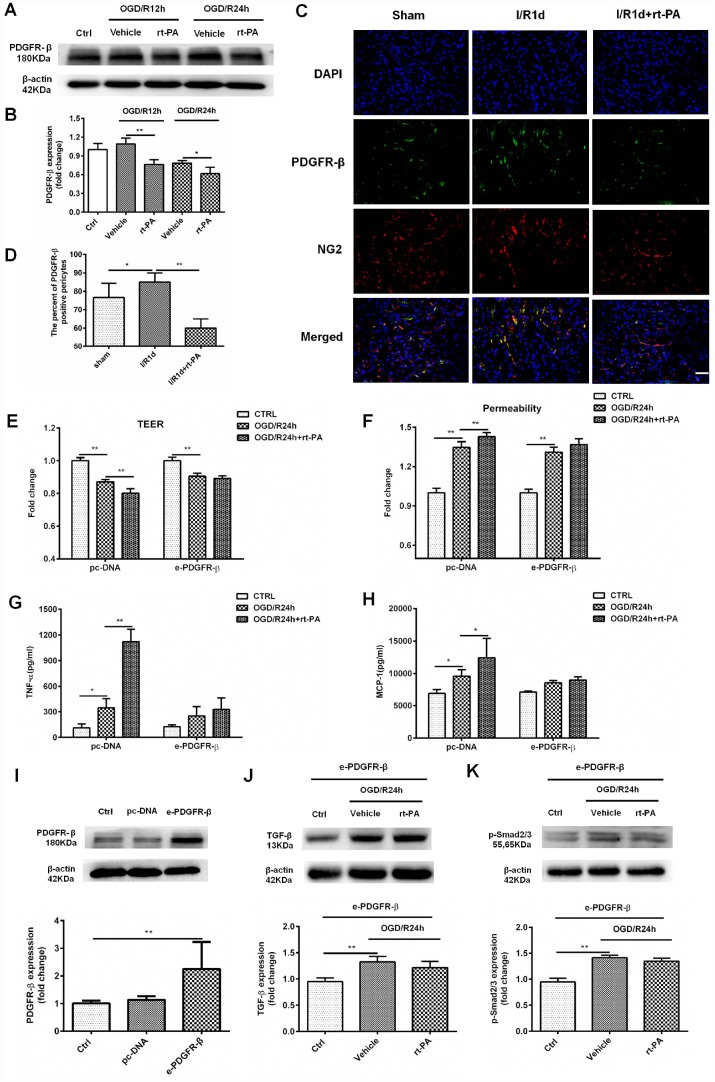
**PDGFR-β mediated the disruption of the BBB after rt-PA treatment.** (**A**, **B**) Representative western blot showing the expression of PDGFR-β 12 h and 24 h after treatment with or without 50 μg/ml rt-PA following OGD for 4 h. Densitometric analysis showing that the level of PDGFR-β protein 12 h and 24 h after treatment with or without rt-PA after OGD for 4 h; n = 3 for each group. Data represent the mean ± sd, *p < 0.05, **p < 0.01. (**C**, **D**) Immunofluorescence was used to detect the expression of PDGFR-β in the sham-treated mice and mice treated with or without 9 mg/kg rt-PA 1 d after I/R; scale bar: 50 μm; n = 3 for each group. Data represent the mean ± sd, *p < 0.05, **p < 0.01. (**E**, **F**) The TEER and permeability were measured in the pc-DNA- or e-PDGFR-β-transfected pericytes cocultured with endothelial cells alone or in the presence of 50 μg/ml rt-PA 24 h after OGD/R; n = 3–5 for each group. Data represent the mean ± sd, *p < 0.05, **p < 0.01. (**G**, **H**) The concentrations of TNF-α and MCP-1 were measured in pc-DNA- or m-PDGFR-β-transfected pericytes cocultured with endothelial cells alone or in the presence of 50 μg/ml rt-PA 24 h after OGD/R; n = 3 for each group. Data represent the mean ± sd, *p < 0.05, **p < 0.01. (**I**) The expression of PDGFR-β in the control, pc-DNA-, and m-PDGFR-β-transfected pericytes was determined by western blotting; n = 3 for each group. Data represent the mean ± sd, *p < 0.05, **p < 0.01. (**J**, **K**) The expression of TGF-β and p-Smad2/3 was measured in PDGFR-β-overexpressing pericytes treated with or without 50 μg/ml rt-PA 24 h after OGD/R; n = 3 for each group. Data represent the mean ± sd, *p < 0.05, **p < 0.01.

PDGFR-β signaling plays an important role in BBB functional recovery and integrity after cerebral ischemia. To determine whether the rt-PA-induced disruption of the BBB is mediated by PDGFR-β on pericytes under hypoxic conditions, we used western blotting to measure the expression of PDGFR-β in control cells and cells subjected to OGD and reoxygenation for 12 h or 24 h with or without rt-PA treatment. Rt-PA decreased the expression of PDGFR-β compared with that following OGD/R alone (Figure 4A, 4B). To further test this hypothesis, we performed double-labeling immunofluorescence in mice subjected to I/R for 1 d treated with or without 9 mg/kg rt-PA. The results of this experiment also showed that rt-PA treatment decreased PDGFR-β (green) colocalization with NG2 (red) compared with that following I/R alone for 1 d (Figure 4C, 4D). To further study whether the rt-PA-induced disruption of the BBB is mediated by PDGFR-β, we first overexpressed PDGFR-β in pericytes using e-PDGFR-β, and the expression of PDGFR-β was determined by western blotting. As shown in Figure 4I, PDGFR-β expression was increased by 2.25 ± 0.9-fold following e-PDGFR-β transfection compared with that following pc-DNA transfection. Then, we measured the TEER and permeability in pc-DNA- and e-PDGFR-β-transfected pericytes cocultured with endothelial cells after OGD and reoxygenation for 24 h alone or in the presence of 50 μg/ml rt-PA. Rt-PA did not decrease the TEER and increase the permeability compared with those following OGD and reoxygenation alone for 24 h in e-PDGFR-β-overexpressing pericytes cocultured with endothelial cells; however, the TEER was decreased and the permeability was increased in pc-DNA-transfected pericytes cocultured with endothelial cells (Figure 4E, 4F). These data suggested that the rt-PA-induced disruption of the BBB is mediated by PDGFR-β on pericytes after I/R. We also studied whether PDGFR-β mediates TNF-α and MCP-1 secretion after rt-PA administration. We measured the concentrations of TNF-α and MCP-1 in pc-DNA- and e-PDGFR-β-transfected pericytes after OGD and reoxygenation for 24 h alone or in the presence of 50 μg/ml rt-PA. Rt-PA did not increase the concentrations of TNF-α or MCP-1 compared with those following OGD/R but increased the TNF-α and MCP-1 levels in pc-DNA-transfected pericytes (Figure 4G, 4H). A previous study indicated that PDGFR-β deletion decreases TGF-β expression after ischemic stroke [19]; thus, we aimed to determine whether PDGFR-β affects TGF-β expression after rt-PA administration. Then, we measured the expression levels of TGF-β and p-Smad2/3 in PDGFR-β-overexpressing pericytes after OGD and reoxygenation for 24 h with or without rt-PA treatment (Figure 4J, 4K). Rt-PA administration did not decrease the expression levels of TGF-β and p-Smad2/3 in PDGFR-β-overexpressing pericytes compared with those following OGD and reoxygenation alone for 24 h. Our results indicated that PDGFR-β might mediate the expression of TGF-β and p-Smad2/3 after rt-PA administration.

### Reduced TGF-beta signaling mediates rt-PA-induced BBB disruption

**Figure 5:**
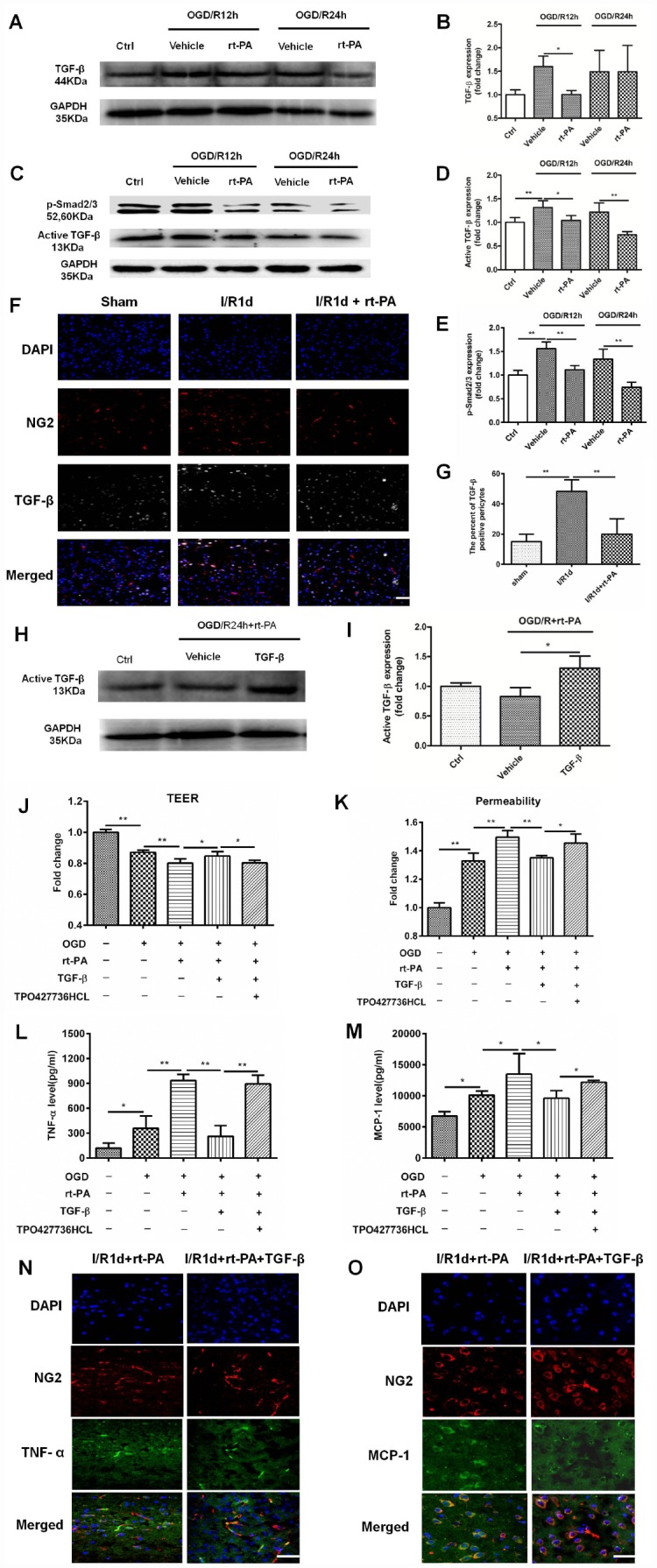
**The TGF-β/p-Smad2/3 pathway mediated the disruption of the BBB after rt-PA treatment.** (**A**–**E**) Representative western blot showing the expression of total TGF-β (44 kDa), active TGF-β (13 kDa) and p-Smad2/3 at 12 h and 24 h after treatment with or without 50 μg/ml rt-PA after OGD for 4 h. Densitometric analysis of the levels of total TGF-β (44 kDa), active TGF-β (13 kDa) and p-Smad2/3 protein at 12 h and 24 h after treatment with or without rt-PA after OGD for 4 h; n = 3 for each group. Data represent the mean ± sd, *p < 0.05, **p < 0.01. (**F**, **G**) Immunofluorescence was used to detect the expression of TGF-β on pericytes in the sham-treated mice and mice 1 d after I/R treatment with or without 9 mg/kg rt-PA; scale bar: 50 μm; n = 3 for each group. Data represent the mean ± sd, *p < 0.05, **p < 0.01. (**H**, **I**) Representative western blot showing the expression of active TGF-β (13 kDa) after treatment with TGF-β in combination with rt-PA or rt-PA alone after OGD for 4 h. Densitometric analysis of the level of active TGF-β (13 kDa) after treatment with TGF-β in combination with rt-PA or rt-PA alone after OGD for 4 h; n = 3 for each group. Data represent the mean ± sd, *p < 0.05, **p < 0.01. (**J**, **K**) The TEER and permeability of the coculture model were determined after OGD/R alone or OGD/R in combination with 50 μg/ml rt-PA, 3 ng/ml TGF-β or 200 nM TPO427736 HCL treatment; n = 3–5 for each group. Data represent the mean ± sd, *p < 0.05, **p < 0.01. (**L**, **M**) The concentrations of TNF-α and MCP-1 secreted from pericytes alone or treated with 50 μg/ml rt-PA, 3 ng/ml TGF-β or 200 nM TPO427736 HCL were determined 24 h after OGD/R; n = 3–4 for each group. Data represent the mean ± sd, *p < 0.05, **p < 0.01. (**N**, **O**) Immunofluorescence was used to detect the expression of TNF-α and MCP-1 after 1 d of treatment with 10 μg/kg TGF-β in combination with 9 mg/kg rt-PA after I/R. The results also showed that TGF-β decreased the colocalization of TNF-α and MCP-1 (green) with NG2 (red) compared with rt-PA alone.

TGF-β is an anti-inflammatory factor [26], and a previous study suggested that the TGF-β pathway can suppress MCP-1 secretion from pericytes [9]. To assess whether reductions in TGF-beta signaling mediate rt-PA induced inflammatory response after rt-PA administration, we first measured the expression of TGF-β and p-Smad2/3 after OGD and reoxygenation for 12 h and 24 h with and without 50 μg/ml rt-PA treatment. The change in total TGF-β (44 kDa) expression was significant after rt-PA treatment for 12 h but not significant after rt-PA treatment for 24 h. Rt-PA decreased the expression of active TGF-β (13 kDa) and p-Smad2/3 compared with OGD/R alone (Figure 5A–5E). To further confirm this result, we performed double-labeling immunofluorescence 1 d after I/R with or without 9 mg/kg rt-PA treatment. The results of this experiment also showed that rt-PA decreased the colocalization of TGF-β (green) with NG2 (red) compared with that 1 d after I/R alone (Figure 5F, 5G). To determine whether the TGF-β levels increased to control levels or higher following this treatment, we measured the active TGF-β (13 kDa) levels after treatment with TGF-β in combination with rt-PA or rt-PA alone under hypoxic conditions. The results indicated that TGF-β in combination with rt-PA treatment increased the active TGF-β level compared with rt-PA treatment under hypoxic conditions (Figure 5H, 5I). To assess whether disruption of the BBB by rt-PA is mediated by TGF-β-p/Smad2/3, we measured the TEER and permeability in an *in vitro* BBB model subjected to OGD/R with 50 μg/ml rt-PA, 3 ng/ml TGF-β or 200 nM TPO427736 HCL treatment. TGF-β increased the TEER and decreased the permeability compared with those following rt-PA treatment in the coculture model, and these changes were abrogated by cotreatment with TPO427736 HCL (Figure 5J, 5K). To investigate whether the TGF-β pathway, which protects the BBB, is associated with TNF-α and MCP-1 generation after rt-PA administration, we measured TNF-α and MCP-1 secretion from pericytes after reoxygenation for 12 h and 24 h with 50 μg/ml rt-PA, 3 ng/ml TGF-β or 200 nM TPO427736 HCL treatment after 4 h of OGD. TGF-β decreased TNF-α and MCP-1 secretion compared with that following rt-PA treatment, and this effect was also abrogated by cotreatment with TPO427736 HCL (Figure 5L, 5M). Double-labeling immunofluorescence was performed 1 d after I/R treatment with 10 μg/kg TGF-β in combination with 9 mg/kg rt-PA. The results also showed that TGF-β decreased the colocalization of TNF-α and MCP-1 (green) with NG2 (red) compared with rt-PA alone (Figure 5N, 5O)

## DISCUSSION

Cerebral ischemia is the major cause of mortality and the leading cause of long-term disability. Treatment with rt-PA is an important medical therapy after ischemic stroke. Rt-PA is known to have a deleterious effect on the BBB and can induce the increase in the incidence of intracerebral hemorrhage [27, 28]. Our results indicated that rt-PA treatment increased the brain water content and increased the disruption of the BBB ultrastructure compared with I/R treatment.

Multiple mechanisms to explain the deleterious effect of rt-PA on NVU permeability have been reported. These mechanisms involve the rt-PA-LRP pathway [29], rt-PA-PDGF-CC pathway [30], rt-PA-MMP-9 pathway [31], rt-PA-NMDA receptor pathway [32], and rt-PA–APC/PAR1 pathway [33], but the specific mechanism underlying the effect of rt-PA on pericytes is lacking. Pericytes, a very important component of the BBB, play many important roles. When cerebral infarction occurs, many pericytes migrate to the area around the ischemic necrosis; this phenomenon is most likely related to repair and vascular remodeling in the infarct brain tissue [34]. Therefore, we wanted to determine whether pericytes play an important role in BBB disruption after rt-PA administration. The results suggested that rt-PA significantly decreases pericyte survival and proliferation. A previous study also demonstrated that rt-PA is toxic to pericytes [12]. In addition, our results showed that rt-PA disrupted the integrity of an *in vitro* coculture model. Studies have suggested that TNF-α and MCP-1 disrupt the BBB [5, 6]. A previous study also indicated that pericytes could generate TNF-α and MCP-1 after monomeric α-synuclein treatment [14]. The inflammatory response occurs early, and thus, we determined the expression of TNF-α and MCP-1 after 12 h and 24 h of reoxygenation following OGD for 4 h. The administration of rt-PA increased TNF-α and MCP-1 secretion from the pericytes under hypoxia condition. These findings are consistent with a previous study that reported the proinflammatory effect of rt-PA after ischemic stroke [7]. Thus, understanding the mechanisms of this effect may lead to the discovery of an effective neuroprotective strategy for the treatment of acute ischemic stroke.

Many studies have indicated that TGF-β plays a protective role in BBB integrity. A previous study suggested that TGFβ is part of the anti-inflammatory response [26]. Here, rt-PA administration decreased the expression of TGF-β on pericytes, and our data also indicated that rt-PA treatment decreased the expression of p-Smad2/3. Our results further suggested that TGF-β administration increased the TEER and decreased the permeability compared with those following rt-PA treatment and that this effect was abrogated by cotreatment with TPO427736 HCL. The results of our study are consistent with the finding that TGF-β derived from pericytes could strengthen BBB barrier integrity in an *in vitro* BBB model [19]. Our data are also consistent with the role of TGF-β/Smad2/3 signaling in regulating BBB integrity [19]. In contrast, our results are in conflict with the finding that TGF-β1 enhances the expression of classical proinflammatory cytokines and enzymes that disrupt BBB functioning [35]. This discrepancy may be explained by the doses of TGF-β1 and the different cell lines used in different studies. The TGF-β/Smad2/3 pathway can modify inflammatory protein secretion in human brain pericytes [9, 36]. Our findings also suggested that TGF-β decreases TNF-α and MCP-1 secretion from pericytes after treatment with rt-PA, and this change was abrogated by cotreatment with TPO427736 HCL. These results are consistent with the finding that the TGF-β pathway can suppress MCP-1 secretion from pericytes [9].

PDGFR-β, a cytoplasmic protein that helps maintain the integrity of the BBB, is specifically expressed in pericytes. The expression of PDGFRβ is increased after cerebral ischemia [37, 38]. Western blotting and immunofluorescence analysis showed that rt-PA decreased the expression of PDGFR-β. Furthermore, rt-PA treatment did not decrease the expression of TGF-β or Smad2/3 in PDGFR-β-overexpressing pericytes after OGD/R. These results are consistent with the effects of PDGFR-β deletion, which resulted in decreased TGF-β expression after ischemic stroke [19]. We found that rt-PA treatment did not decrease the TEER or increase the permeability of PDGFR-β-overexpressing pericytes in an endothelial cell coculture model. Our data agree with that the permeability of ^14^C sucrose increased in si-PDGFR-β-transfected pericytes in the endothelial coculture model and that exogenous TGF-β1 partly decreased ^14^C sucrose permeability, while SB431542 reversed this effect [19]. We also found that rt-PA treatment did not increase TNF-α or MCP-1 secretion from PDGFR-β-overexpressing pericytes.

Our finding has important clinical implications. Many studies had reported combined therapy could attenuate the brain damage and BBB disruption in the presence of rt-PA [8, 39]. In this study, we first study the inflammation response of pericytes after hypoxia. Besides that we first study rt-PA disrupt BBB by increasing the expression of inflammation factors in the pericytes. Based on our observations, we propose a PDGFR-β/TGF-β/p-Smad2/3 pathway to explain the deleterious effect of rt-PA on the permeability of the NVU *in vitro* and *in vivo*. At this time, this signaling axis mediating the effect of rt-PA on the BBB through its influence on pericytes is unique. Therefore, this finding has important clinical implications for not only further elucidating the effect of rt-PA on BBB disruption but also developing an effectively combined therapy for the administration of rt-PA.

Our study has several limitations. First, the *in vitro* BBB model cannot absolutely mimic the BBB *in vivo*. Furthermore, we did not study the mice for a long period of time after I/R based on their mortality. Finally, the mechanism reported in this study needs to be further tested *in vivo*.

## MATERIALS AND METHODS

### Animal models

Eight-to-ten-week-old male C57BL/6 mice weighing 20–30 g were used in the experiment. Mice were randomly assigned to each group. C57BL/6 mice were subjected to left MCAO for 1 h followed by reoxygenation for 1 d. MCAO was performed via inserting a nylon monofilament (Jialing, China) into the left internal carotid artery as previously described [40]. The mice were intravenously injected with etanercept (200 μg/kg) or TGF-β1 (10 μg/kg) at the moment of reperfusion, and rt-PA (9 mg/kg) was intravenously injected 1 h after reperfusion. We selected the dosage of rt-PA based on previous studies [41]. Laser-Doppler flowmetry was applied to monitor cerebral blood flow during MCAO. A heating pad was used to maintain the temperature of the mice at 37°C throughout the process. Mice in the sham group were anesthetized, and the left carotid bifurcation was exposed, but the filament was not inserted. Mice were included when the cerebral blood flow (CBF) was < 30% of basic blood flow levels throughout the ischemic period and the recovery of CBF was >80% within 10 min of reperfusion. All animal experiments were approved by the Tianjin Huanhu Hospital Laboratory Animal Center. The animal experimental data reported in this study are in compliance with the ARRIVE (Animal Research: Reporting *in vivo* Experiments) guidelines.

### Measurement of brain edema

The brains of mice under deep intraperitoneal anesthesia were harvested, cut into contralateral and ipsilateral hemispheres and then weighed to record their wet weights. The brain tissues were placed in a 50°C incubator for 72 h, and their dry weights were recorded. The brain water content was calculated as previously reported [42] with the following formula: %H_2_O = (wet weight − dry weight)/wet weight * 100%.

### Transmission electron microscopy

The mice were rapidly perfused with 4% paraformaldehyde through the heart. Each brain was harvested, and 1 mm^3^ pieces were cut from the ischemic cerebral cortex and fixed in 2% glutaraldehyde. Conventional transmission electron microscopy samples were prepared by semithin sectioning, ultrathin sectioning, and uranium and lead double staining, and the samples were then observed and photographed with an H-7760 microscope.

### Leakage of FITC-dextran

BBB damage was determined by assessing the leakage of fluorescein isothiocyanate-conjugated dextran (FITC-dextran, 70 kDa, Sigma). A total of 0.3 ml of 5% FITC-dextran was injected through the femoral vein 3 min before the brain was harvested. The mice were transcardially perfused with ice-cold PBS, followed by immersion in 4% paraformaldehyde. Coronal sections were prepared, blocked and then cut. Frozen sections were photographed with a confocal microscope (TCS SP5, Leica).

### Cell culture

Mouse brain microvascular pericytes (Beinachuanglian, China) and brain microvascular endothelial cells (Beinachuanglian, China) were cultured in Dulbecco's modified Eagle's medium (DMEM, Gibco, USA) containing 10% fetal bovine serum (S711-001, Lonza Science SRL), 100 units/ml penicillin and 100 mg/ml streptomycin. Pericytes and endothelial cells were cultured in humidified air containing 5% CO2 and 95% atmosphere at 37°C.

### Construction of *in vitro* BBB models

The pericytes and endothelial cells were used to construct *in vitro* BBB models when they reached 70–80% confluency as previously reported [43]. Pericytes (15000 cells/cm^2^) were seeded on the outside of 24-well Transwell inserts (0.33 cm^2^, 0.4 mm pore size) coated with collagen polycarbonate membrane for 6 h and then turned upside down in the 24-well culture plate (Corning, USA). The endothelial cells (150000 cells/cm^2^) were seeded on the inside of the Transwell insert after the pericytes had been cultured for 1 d. The coculture medium (0.3 ml within the insert and 0.9 ml in the outer well) was replaced every two days.

### Cell transfection

Pericytes were transfected with e-PDGFR-β and pc-DNA (according to the manufacturer’s instructions). Pc-DNA and e-PDGFR-β were diluted to 10 ng/μl in Opti-MEM medium (Life Technologies, Carlsbad, CA, USA), Lipofectamine 3000 and P3000 transfection reagent (11668, Life Technologies). The diluted mixture was incubated for 10 min at room temperature and then added to the outer well of the *in vitro* BBB model for 48 h before the cells were used for subsequent experiments. For determination of e-PDGFR-β transfection efficiency, total protein was extracted for western blot analysis after 48 h of transfection, and pc-DNA transfection was used as a control.

### OGD/R

Pericytes and the *in vitro* BBB model were exposed to OGD/R. Briefly, the culture medium was replaced with glucose-free DMEM (Gibco, USA) and incubated in a hypoxic chamber (Thermo Billups Rothenberg, CA, USA) containing 5% CO2, 1% O2 and 94% N2 at 37°C. After 4 h of OGD, glucose and pyruvate were added to the culture medium, and the cells were grown under normal culture conditions. TGF-β (3 ng/ml), TPO427736HCL (200 nM), etanercept (40 μg/ml) or rt-PA (50 μg/ml) was administered at the moment of reoxygenation. We used the dosage of rt-PA according to as previously described [12].

### TEER and permeability assays

The integrity of the *in vitro* BBB models was evaluated by measuring the TEER and dextran permeability as described previously [44]. An EVOM2 epithelial volt-ohm meter and an STX2 electrode set (World Precision Instruments) were used to measure the TEER. The TEER of the blank filter was subtracted from the TEER of all groups to obtain the final results. The TEER is shown as Ω/cm^2^ and was calculated with the following formula: TEER (Ω/cm^2^) = TEER (Ω) × surface area (0.33 cm^2^). The permeability of the *in vitro* BBB model was assessed through analyzing fluxes in the fluorescence of FITC-conjugated dextran (MW 40000 Da; Sigma) as previously described [45, 46]. Briefly, 100 μg/ml FITC-dextran in DMEM was added to the inner chamber of the *in vitro* BBB model to measure permeability. At 0, 30, 60, and 120 min after FITC-conjugated dextran had been added, 50 μl of culture medium was removed from the outer chamber, and an equal volume of assay buffer was added to the well. The medium was analyzed by a fluorescence microplate reader (excitation wavelength, 485 nm; emission wavelength, 525 nm). The permeability to dextran after different treatments is shown as a percentage of that of the control. The permeability coefficient was calculated as follows: permeability coefficient (cm/min) = V/(SA * Cd) * (Cr/T), where V is the volume of medium in the outer chamber, SA is the area of the membrane, Cd is the concentration of FITC-conjugated dextran in the inner chamber at time 0, and Cr is the concentration of FITC-conjugated dextran in the outer chamber at sampling time T. All data were collected from at least 3 independent experiments.

### MTT assay

For determination of the OGD time, the pericyte survival rate was determined after OGD for 2 h, 4 h and 6 h. For analysis the effect of rt-PA on pericytes, the pericyte survival rate was determined 12 h and 24 h after OGD for 4 h and reoxygenation alone or in combination with rt-PA at 50 μg/ml. MTT solution was added to pericytes seeded on 24-well plates, which were incubated for 4 h at 37°C. The resultant formazan was dissolved in DMSO, and the absorbance was then measured at a wavelength of 490 nm.

### RT-QPCR

Pericyte RNA was extracted using an HP Total RNA Kit (Omega, R681l-2) according to the manufacturer’s instructions. Then, RNA was reverse transcribed to cDNA using a SureFireRT Kit (AIBOJIN, 06-104). RT-QPCR was performed using a Roche 480 instrument. The primers used were as follows: mouse TNF-α, sense 5′-CATCTTCTCAAAATTCGAGTGACAA-3′ mouse TNF-α, antisense 5′-TGGGAGTAGACAAGGTACAA CCC-3′ mouse MCP-1, sense 5′-GAAGGAATGGG TCCAGACAT-3′ mouse MCP-1, antisense 5′-ACGG GTCAACTTCACATTCA-3′ mouse IL-1β, sense 5′-AG AAGCTGTGGCAGCTA-3′ mouse IL-1β, antisense 5′-TGAGGTGCTGATGTACCA-3′ mouse GAPDH, sense 5′-TTCACCACCATGGAGAAGGC-3′ mouse GAPDH, antisense 5′-GGCATGGACTGTGGTCATGA-3′.

### ELISA

To quantify MCP-1 and TNF-α secreted from pericytes after different treatments, we used mouse MCP-1 and TNF-α solid-phase sandwich ELISAs (Cloud-Clone Corp.). Cell culture media were collected and centrifuged at 1000 × g at 4°C for 20 min to discard the cellular debris. Then, the levels of selected cytokines and chemokines in the supernatants were determined following the manufacturer’s instructions.

### Western blot analysis

The total protein was extracted after different treatments. Protein samples (20 μg) were separated on 8–15% gradient gels (ATTO, Tokyo, Japan), transferred to nitrocellulose membranes (ATTO) and blocked with 5% BSA at room temperature for 2 h. The membranes were incubated at 4°C overnight with the following primary antibodies: mouse polyclonal anti-GAPDH (1:5000, Proteintech), mouse polyclonal anti-β-actin (1:5000, Proteintech) rabbit polyclonal anti-PDGFR-β (1:500, Proteintech), rabbit polyclonal anti-TGF-β (13 kDa,1:500, Wanlei), rabbit polyclonal anti-TGF-β (44 kDa,1:500, Proteintech), and mouse monoclonal anti-p-Smad2/3 (1:500, Wanlei). The membranes were washed with TBST and then incubated with secondary antibodies for 1 h at room temperature. Protein bands were analyzed by ImageJ software after the membranes had been carefully washed with TBST.

### Immunofluorescence staining of frozen tissue sections

Mice under deep anesthesia were transcardially perfused with ice-cold PBS, followed by immersion in 4% paraformaldehyde. Coronal sections were prepared, blocked and then cut. Frozen sections were incubated overnight at 4°C with the following primary antibodies: rabbit polyclonal anti-PDGFR-β (1:50, Cell Signaling Technology), mouse monoclonal anti-NG2 (a marker of cerebrovascular pericyte 1:100, Santa Cruz), rabbit polyclonal anti-TGF-β (1:100, Abcam), rabbit polyclonal anti-Ki-67 (1:100, Cell Signaling Technology), rabbit polyclonal anti-MCP-1 (1:200, Abcam) and rabbit polyclonal anti-TNF-α (1:50, Abcam). Then, the sections were incubated with appropriate host secondary antibodies conjugated with Alexa Fluor 488, Alexa Fluor 594 or Alexa Fluor 637 (1:200, Invitrogen, USA). All sections were stained with 6-diamidino-2-phenylindole (DAPI, CA, USA) and photographed with a confocal microscope (TCS SP5, Leica).

### Statistical analysis

The statistical significance of differences among the groups was analyzed via one-way ANOVA using the SPSS 22.0 software package. Differences were considered statistically significant at *p* < 0.05. All results are expressed as the mean ± sd.
